# Supplementary material for: Development of Type 1 Diabetes in Mice Is Associated with a Decrease in IL-2-Producing ILC3 and FoxP3+ Treg in the Small Intestine
Source: Molecules. 2023 Apr 11;28(8):3366. doi: 10.3390/molecules28083366 (PMC10141349; doi:10.3390/molecules28083366)
Supplement: Supplementary file 1 [file molecules-28-03366-s001.zip › molecules-2242454-supplementary.pdf]

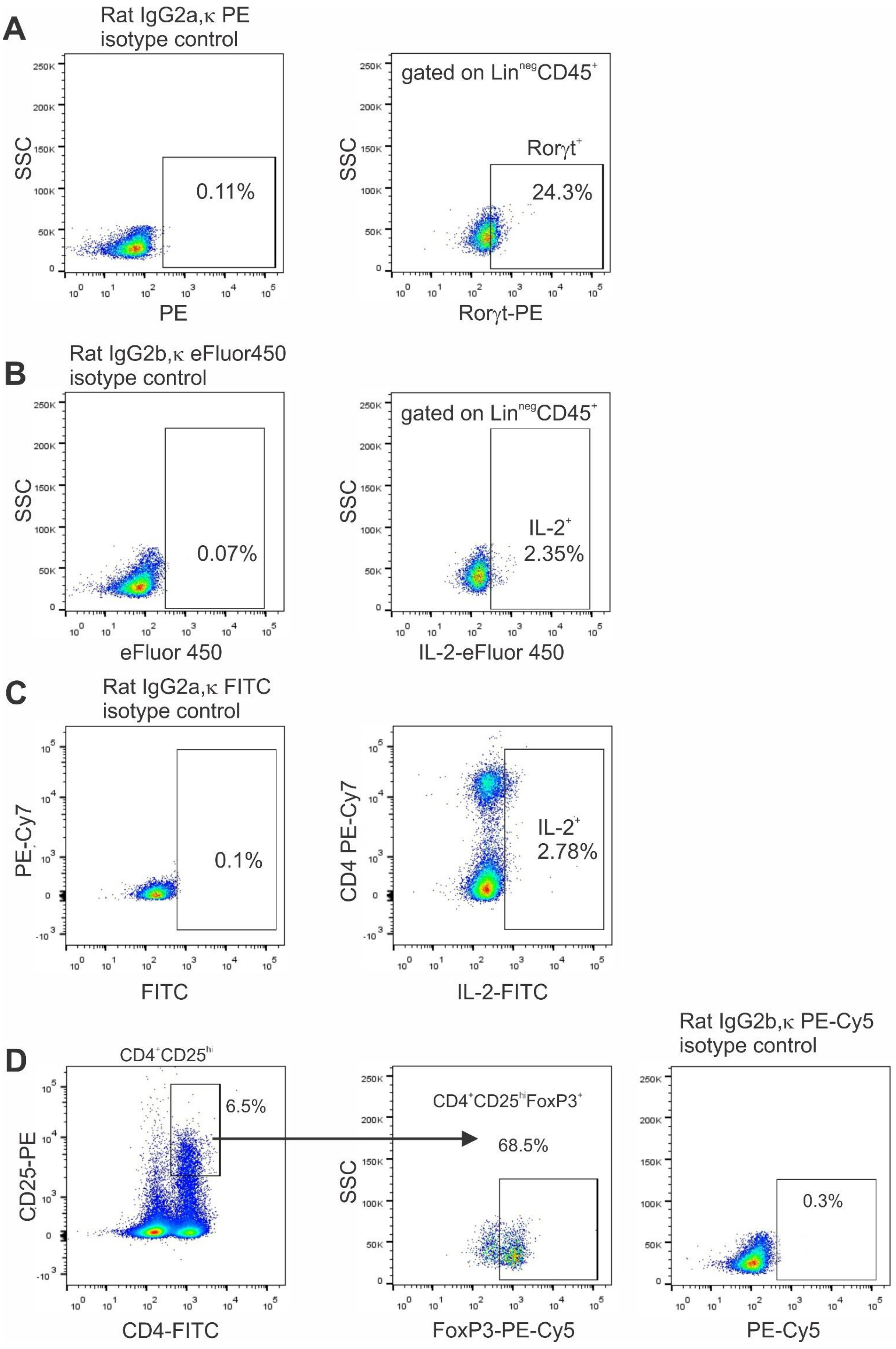

A
Rat IgG2a,κ PE
isotype control
SSC
PE
0.11%
gated on Lin^neg CD45^+
Rorγt^+
24.3%
Rorγt-PE
B
Rat IgG2b,κ eFluor450
isotype control
eFluor 450
0.07%
IL-2^+
2.35%
IL-2-eFluor 450
C
Rat IgG2a,κ FITC
isotype control
PE-Cy7
FITC
0.1%
CD4 PE-Cy7
IL-2^+
2.78%
IL-2-FITC
D
CD4^+CD25^hi
6.5%
CD25-PE
CD4-FITC
CD4^+CD25^hi FoxP3^+
68.5%
FoxP3-PE-Cy5
Rat IgG2b,κ PE-Cy5
isotype control
0.3%
PE-Cy5

Figure S1. Isotype controls and gating strategy. A) Rorγt PE staining and the adequate isotype control, SILP cells. B) IL-2 eFluor450 staining and the adequate isotype control, SILP cells (for the experiment where comparison was made between healthy and MLDS hyperglycemic C57BL/6 mice. C) IL-2 FITC within $CD4^+$ and the adequate isotype control, spleen cells (for the experiment where comparison was made between young and old NOD and MLDS and ABX+MLDS mice. D) Gating strategy for Treg (spleen cells) and the adequate isotype control for FoxP3.
